# Artificial Intelligence for Cardiac Imaging-Genetics Research

**DOI:** 10.3389/fcvm.2019.00195

**Published:** 2020-01-21

**Authors:** Antonio de Marvao, Timothy J. W. Dawes, Declan P. O'Regan

**Affiliations:** MRC London Institute of Medical Sciences, Imperial College London, London, United Kingdom

**Keywords:** artificial intelligence, machine learning, deep learning, genetics, genomics, imaging-genetics, cardiovascular imaging, cardiology

## Abstract

Cardiovascular conditions remain the leading cause of mortality and morbidity worldwide, with genotype being a significant influence on disease risk. Cardiac imaging-genetics aims to identify and characterize the genetic variants that influence functional, physiological, and anatomical phenotypes derived from cardiovascular imaging. High-throughput DNA sequencing and genotyping have greatly accelerated genetic discovery, making variant interpretation one of the key challenges in contemporary clinical genetics. Heterogeneous, low-fidelity phenotyping and difficulties integrating and then analyzing large-scale genetic, imaging and clinical datasets using traditional statistical approaches have impeded process. Artificial intelligence (AI) methods, such as deep learning, are particularly suited to tackle the challenges of scalability and high dimensionality of data and show promise in the field of cardiac imaging-genetics. Here we review the current state of AI as applied to imaging-genetics research and discuss outstanding methodological challenges, as the field moves from pilot studies to mainstream applications, from one dimensional global descriptors to high-resolution models of whole-organ shape and function, from univariate to multivariate analysis and from candidate gene to genome-wide approaches. Finally, we consider the future directions and prospects of AI imaging-genetics for ultimately helping understand the genetic and environmental underpinnings of cardiovascular health and disease.

## Introduction

Cardiovascular conditions remain the leading cause of mortality and morbidity worldwide (1), with genetic factors playing a significant role in conferring risk for disease (2). High-throughput DNA sequencing and genotyping technologies, such as whole-genome sequencing and high-resolution array genotyping, have developed at an extraordinary pace since the first draft of the human genome was published in 2001 at a cost of $0.5-1 billion (3). Continuous improvements have so far outpaced Moore's law, with the sequencing cost per genome currently estimated to be $1,000 (4), enabling cost-effective sequencing of millions of humans. At the same time, technological advances in physics, engineering, and computing have enabled a step-change improvement in cardiovascular imaging, facilitating the shift from one dimensional, low-fidelity descriptors of the cardiovascular system to high-resolution multi-parametric phenotyping. These capabilities are not limited to research settings but are increasingly available in clinical echocardiography, nuclear imaging, computerized tomography (CT), and cardiovascular magnetic resonance (CMR) practice. An unprecedented volume of clinical data is also becoming available, from smartphone-linked wearable sensors (5) to the numerous variables included in the electronic health records of entire populations (6). However, the volume, heterogeneity, complexity, and speed of accumulation of these datasets now make human-driven analysis impractical. Artificial intelligence (AI) methods such as machine learning (ML), are particularly suited to tackling the challenges of “Big Data” and have shown great promise in addressing complex classification, clustering, and predictive modeling tasks in cardiovascular research. Cardiac imaging-genetics refers to the integrated research methods that aim to identify and characterize the genetic variants that influence functional, physiological, and anatomical phenotypes derived from cardiovascular imaging.

In the same way that basic statistical literacy has become a routine aspect of clinical practice, a basic understanding of AI's strengths, applications, and limitations is becoming essential for practicing researchers and clinicians. Here we introduce common AI principles, review applications in imaging-genetics research, and discuss future directions and prospects in this field.

## Imaging-Genetics: From Single Gene Hypothesis-Testing to Genome-Wide Hypotheses Generation

Imaging-genetics aims to dissect and characterize the complex interplay between imaging-derived phenotypes and environmental and genetic factors. Many principles and approaches originated from neuroimaging research, where the first attempts at integrating multi-parametric phenotypes, obtained from structural and functional brain MRI, with genetic data were carried out (7). To help manage the computational and statistical challenges inherent to the use of “Big Data” squared (high-dimensional imaging × high-dimensional genetic data), interrogations were limited to pre-defined regions of interest in the brain and candidate genes or SNPs, based on *a priori* assumptions about the biology of disease (8). Similar, “hypothesis-led” designs underpinned candidate gene and linkage studies that established causal relationships between rare genetic variants and rare conditions, such as those that first identified the role of myosin heavy-chain beta in hypertrophic cardiomyopathy (HCM) (9) and of titin in dilated cardiomyopathy (DCM) (10).

The increased affordability of DNA sequencing and genotyping resulted in genetic information becoming available in large numbers of subjects. This has contributed to shift the focus to genetic discovery and the study of common, complex disease traits. These traits are not characterized by a single gene mutation leading to a large change on the phenotype but attributable to the cumulative effects of many loci. Although the effect sizes of individual loci are relatively modest, composite effects can significantly alter the probability of developing disease (11). The “common disease—common variant” hypothesis underpins genome wide association studies (GWAS), where subjects are genotyped for hundreds of thousands of common variants. For example, a study into the genetic determinants of hypertension in over 1 million subjects, identified 901 loci that were associated with systolic blood pressure (SBP) and these explained 5.7% of the variance observed (12). Even though these single nucleotide polymorphisms (SNPs) explain only a small proportion of phenotypic variance they provide relevant, hypothesis-generating biological or therapeutic insights. The rapid development of complementary high-throughput technologies, able to characterize the transcriptome, epigenome, proteome, and metabolome now enables us to search for molecular evidence of gene causality and to understand the mechanisms and pathways involved in health and disease (13). These large biological multi-omics data sets and their computational analysis are conceptually similar to the more established study of genomics and examples of such work are included in this review.

## Imaging-Genetics: From One-Dimensional Phenotyping to Multiparametric Imaging

Several biological and technical reasons have been proposed to explain the “missing heritability” of complex cardiovascular traits. However, a common factor limiting many genotype-phenotype studies was that the ability to characterize phenotypes rapidly and accurately, significantly lagged behind our ability to describe the human genotype (14). Phenotyping was characterized by imprecise quantification, sparsity of measurements, high intra- and inter- observer variability, low signal to noise ratios, reliance on geometric assumptions, and adequate body habitus, poor standardization of measurement techniques and the tendency to discretize continuous phenotypes (15). Commonly, the complexity of the cardiovascular system was distilled into a small number of continuous one-dimensional variables [e.g. volumetric assessment of the left ventricle (16)] or, convenient dichotomies, such as responders vs. non-responders (17), leading to a loss of statistical power (18).

The imaging community responded to calls for more accurate and precise, high-dimensional phenotyping (19, 20) with the roll out of developments in echocardiography (e.g., tissue doppler, speckle-tracking, and 3D imaging), CMR (e.g., tissue characterization, 4D flow, 3D imaging, diffusion tensor imaging, spectroscopy, and real-time scanning), CT (e.g., improved spatial and temporal resolution, radiation dose reduction techniques, functional assessment of coronary artery flow using FFR-CT, and coronary plaque characterization), and nuclear cardiology (e.g., improvements in radiopharmaceuticals and hardware resulting in increased accuracy and reduced radiation exposure). In parallel, computational approaches have become increasingly integral to the clinical interpretation of these much larger datasets (21–23) and several have obtained FDA approval (24).

## Imaging-Genetics: A “Big Data” Squared Problem

Leveraging these deeper phenotypes is an attractive proposition but the joint analysis of high-dimensional imaging and genetic data poses major computational and theoretical challenges. An early example of a neuroimaging GWAS investigated the association between 448,293 SNPs and 31,622 CMR voxels in a cohort of 740 subjects (25). This study highlighted difficulties correcting for multiple testing (1.4 × 10^10^ tests were performed) and the need for unprecedented computational power (300 parallel cores).

Simultaneously assessing the statistical significance of several hundred thousand tests vastly increases the number of anticipated type I errors. If the probability of incorrectly rejecting the null hypothesis in one test with a pre-set α of 0.05 is 5%, then under the same conditions, the probability of incorrectly rejecting the null hypothesis at least once if 100 tests are performed is 99.4%. Therefore, an adjustment for the number of tests being carried out is required. The simplest approach for adjustment for multiple testing is the Bonferroni correction, where the pre-set α is recalculated as α/m, where m represents the number of independent tests being performed. However, this method is overly conservative when m is large, leading instead to many false negatives. An alternative, extensively-validated method is the Benjamini–Hochberg Procedure (26). Using this approach, instead of controlling for the chance of any false positives, an acceptable maximum fixed percentage of false discoveries (the expected proportion of rejected hypotheses that are false positives) is set.

A further consideration in the statistical analysis of high-dimensional cardiac phenotypes is that a clinically significant signal will not originate from a single voxel but across many voxels in extended, anatomically coherent areas. Indeed, approaches such as threshold-free cluster enhancement (TFCE), which were developed in neuroimaging (27), have recently applied in cardiovascular research (28). Using such methods, both signal size and contiguity with surrounding signal patterns contribute to inference statistics.

## Artificial Intelligence

Artificial intelligence, machine learning, and deep learning are terms that are interlinked, have some overlap but are often incorrectly used interchangeably. AI refers to the overarching field of computer science focused on simulating human cognitive processes. As a subset of AI, machine learning refers to the family of algorithms that share a capacity to perform tasks like classification, regression, or clustering based on patterns or rules iteratively learnt directly from the data without using explicit instructions. ML algorithms can be further subdivided into supervised, unsupervised, and reinforcement learning.

Supervised learning is the most common form of traditional ML and involves the training of models on pairs of input and expected outputs (“labeled” data) and then their deployment to make predictions in previously unseen data. It includes such approaches as nearest neighbor, support vector machines, random forests and naïve Bayes classifiers. Unsupervised learning algorithms are used to address clustering or dimensionality reduction problems by detecting patterns and structures within the data without any prior knowledge or constraints. In other words, the model organizes “unlabeled” data into groupings that share common, previously undefined characteristics. Examples including k-means clustering, t-distributed stochastic neighbor embedding (t-SNE), and association rule learning algorithms. The use of reinforcement learning algorithms (e.g., deep Q networks), common in robotics and gaming applications (29) has now also been trialed in the navigation of 3D datasets for anatomical landmark detection (30).

Deep learning (DL) is a specific ML method inspired by the way that the human brain processes data and draws conclusions. To achieve this, DL applications use a layered structure of algorithms, called an artificial neural network that imitates the biological neural network of the human brain. The word “deep” in “deep learning” refers to the number of layers through which the data is transformed. The most common DL models are convolutional neural networks (CNN), which are extremely efficient at extracting features and often superior to traditional ML in larger, more complex datasets such as medical imaging and genomics (31, 32). However, feature and process interpretability is more amenable in classical ML as even simple DL networks can operate as “black-boxes.” While the computational and time requirements of DL are much higher during training, subsequent inference is extremely fast and DL approaches can be used to accelerate supervised, unsupervised, and reinforcement learning. Indeed, while traditional ML is carried out using central processing units (CPUs), DL was only made possible thanks to the development of graphics processing units (GPUs), which have a massively parallel architecture consisting of thousands of cores and were designed to handle vast numbers of tasks simultaneously.

During the training stage of supervised learning algorithms, the labeled data is divided into training, validation, and testing subsets to reduce overfitting and estimate how well the models generalize. No standard methodologies exist to determine optimum proportions allocated to each set. The training set usually includes a large proportion of the available data and is used for the development of the model. The validation set is used to estimate overall model performance during development and fine-tune the algorithm's hyperparameters (e.g., the number of network layers which could not be learnt). Dividing data into training and validation subsets can be done randomly at the onset of the process or by using a cross-validation approach. This involves dividing the entire dataset into folds of equal size and then training the algorithms in all the folds except one that is left out for validation. The process is repeated until all folds have been used as a validation set and the overall performance of the model is calculated as the average across all validation sets. Finally, an independent (ideally external) test set should be used to assess the model's generalizability.

Despite ML's vast potential and significant performance breakthroughs in fields such as speech recognition, natural language processing, and computer vision, these approaches are not without limitations and vulnerabilities. Some of these are shared with classical statistical approaches (33) while others are entirely novel (34). A significant potential pitfall of ML models derives from the presence of unrecognized confounders that can be present in both the training and testing sets, if they originated from the same dataset. This could result in overfitting of the model to the training data, achieving an artificially inflated performance with poor generalization to other data sets in subsequent studies. The gold-standard approach to address this issue is to obtain a validation dataset acquired by an independent group under real-world conditions. Another possible cause of unsatisfactory generalization of an AI system is if the training data is not an accurate representation of the wider population. For example, an AI model trained on a healthy cohort may not generalize well to a general population that includes extreme disease phenotypes, and a system trained on images from a specific CMR scanner might not perform well when labeling images acquired under different technical conditions. Domain adaptation or transfer learning are fields of AI research that aim to address these challenges.

AI algorithms can also be oversensitive to changes in the input data and therefore vulnerable to unintentional or harmful interference. This was clearly demonstrated in experiments involving “adversarial examples” or inputs that lead the model to make a classification error. For example, the introduction of an imperceptible perturbation in a picture of a benign skin mole resulted in the misclassification as a malignant mole, with 100% confidence (35). The general application of AI has also been hindered by the “black-box” nature of several methodologies. Indeed, full clinical acceptability is only likely if it is possible to explore and scrutinize the predictive features and if the outputs are clinically interpretable.

At a more fundamental level, “Big Data” studies are often no more than observational research. As in classical statistics, observational AI studies cannot test causality and should therefore be considered hypothesis-generating that require further testing. A recent systematic review and meta-analysis of 82 studies applying DL methods to medical imaging found that although the diagnostic performance of DL methods was often reported as equivalent to human experts, few studies tested human vs. DL performance on the same sample and then went on to externally validate their findings (36). Furthermore, apart from a handful of exceptions (37), the effect of AI in routine clinical practice has been rarely tested in the setting of randomized controlled trials. Indeed, it has not been systematically demonstrated that the roll out of AI into clinical practice leads to an improvement in the quality of care, increased efficiency or improved patient outcomes (38). These studies will be required before this technology can be routinely used to help guide clinical care.

Table 1 provides an introduction to some of the technical and methodological aspects that should be considered in AI research.

**Table 1:** Considerations in the use of machine learning in imaging-genetics research.

Selection of AI approach based on clinical question and data characteristics	Supervised methods suited to classification and prediction tasks involving “labeled” data: e.g., image segmentation or survival prediction. Unsupervised methods useful to identify structures and patterns in unlabeled data: e.g., association and clustering. Reinforcement learning algorithms interact with the environment by producing actions that get rewarded or penalized, while identifying the optimal path to address the problem. DL can be used to accelarate supervised, unsupervised or reinforcement learning but is better suited to larger, more unstructured datasets. Classical ML is more likely to work better in smaller training datasets.
Algorithm selection	Are there “off-the-shelf” algorithms tailored to identical problems or validated in similar data? Transparency, understandability and performance are all important features. Try to avoid “black box” approaches where it is not possible to scrutinize the features that inform the classification or explain the outputs in high-stakes decision-making.
Data pre-processing	Several steps are likely to be required in the preparation of data including anonymization, quality control, data normalization and standardization, addressing how to handle missing data points and outliers, imputation of missing values, etc. Is the training data an accurate representation of the wider data/population (e.g., all expected variation present, same technical characteristics)?
Feature selection	A subset of relevant features (variables or predictors) is selected from high dimensional data allowing for a more succinct representation of the dataset.
Data allocation	Evaluate the available data and plan the proportions of data being allocated into the training, testing, and validation datasets. Other approaches include cross validation, stratified cross validation, leave-one-out, and bootstrapping.
Hardware considerations	Based on the volume of data and methodological approaches are CPU clusters, GPUs, or cloud computing better suited?
Evaluation of model performance	Receiver operating characteristic (ROC) curves with accuracy measured by the area under the ROC curve (AUC), C-statistics, negative predictive value, positive predictive values, sensitivity, and specificity, Hosmer–Lemeshow test for goodness of fit, precision, recall, f-measure. Imaging segmentation accuracy (comparison between human expert labels and automated labels) reported as Dice metric, mean contour distance, and Hausdorff distance. If the accuracy is perfect, have too many predictors been included for the sample size or are there confounding biases hidden in the data that may result in the model overfitting the data? Compare performance against standard statistical approaches (i.e., multivariate regression). If several algorithms are tested report on them all and not just on the best performance.
Publication and transparency	Make code and anonymized sample of data publicly available (e.g., GitHub, Docker containers, R packages, or Code Ocean repositories). Encourage independent scrutiny of the algorithm.
Generalization and replication results	Algorithms should be validated by independent researchers on external cohorts and satisfy the requirements of medical devices and software regulatory frameworks.

Nevertheless, the use of machine learning methods in cardiovascular research has grown exponentially over recent years, with an ever increasing set of uses and applications. Traditional supervised ML methods have been applied successfully to classification tasks in extremely diverse input data, ranging from discrimination between sequences underlying *Cis*-regulatory elements from random genome sequences (39), separation of human induced pluripotent stem cell-derived cardiomyocytes of distinct genetic cardiac diseases (CPVT, LQT, HCM) (40) to numerous applications in medical imaging analysis. Examples of this include automated quality control during CMR acquisition (41), high-resolution CMR study of cardiac remodeling in hypertension (42) and aortic stenosis (43), and echocardiographic differentiation of restrictive cardiomyopathy from constrictive pericarditis (44). Unsupervised ML analysis have provided new unbiased insights into cardiovascular pathologies such as by establishing subsets of patients likely to benefit from cardiac resynchronization therapy (45) and by agnostic identification of echocardiography derived patterns in patients with heart failure with preserved ejection fraction and controls (46). Traditional ML has also been used for prediction of outcomes such as hospital readmission due to heart failure (47), survival in pulmonary hypertension (48), and population-based cardiovascular risk prediction (49).

More recently, there has been a greater interest in DL approaches, which have been used with great promise in ever larger-scale classification tasks. Applications include the analysis of CMRs (50), echocardiograms (51), and electrocardiograms (52), identification of the manufacturer of a pacemaker from a chest radiograph (53), aortic pressure waveform analysis during coronary angiography (54); automated categorization of HCM and healthy CMRs (55) and detection of atrial fibrillation using smartwatches (56). DL has also been successfully used to address complex survival prediction tasks in pulmonary hypertension (57) and heart transplantation (58).

The analysis of ever larger and complex genome-scale biological datasets is also particularly suited to ML approaches. One of the strengths of these approaches comes from the ability to discover unknown structures in the data and to derive predictive models without requiring *a priori* assumptions about, frequently poorly understood, underlying biological mechanisms (59). The field is large, diverse and fast moving with new opportunities for AI to synthesize data and optimize the prediction of key functional biological features appearing all the time. Applications of traditional ML have ranged from the prediction of quantitative (growth) phenotypes from genetic data (60), to the identification of proteomic biomarkers of disease (61), to the prediction of metabolomes from gene expression (62). As in cardiology research, there has been growing interest in applying DL to the field of functional genomics. Such approaches have been used to predict sequence specificities of DNA- and RNA-binding proteins (31, 63), transcriptional enhancers (64) and splicing patterns (65) and to identify the functional effects of non-coding variants (66, 67). A more in depth discussion of the applications of ML and DL to genomics and other multi-omics data can be found elsewhere (68–71).

## Artificial Intelligence in Cardiovascular Imaging-Genetics

**Figure 1:**
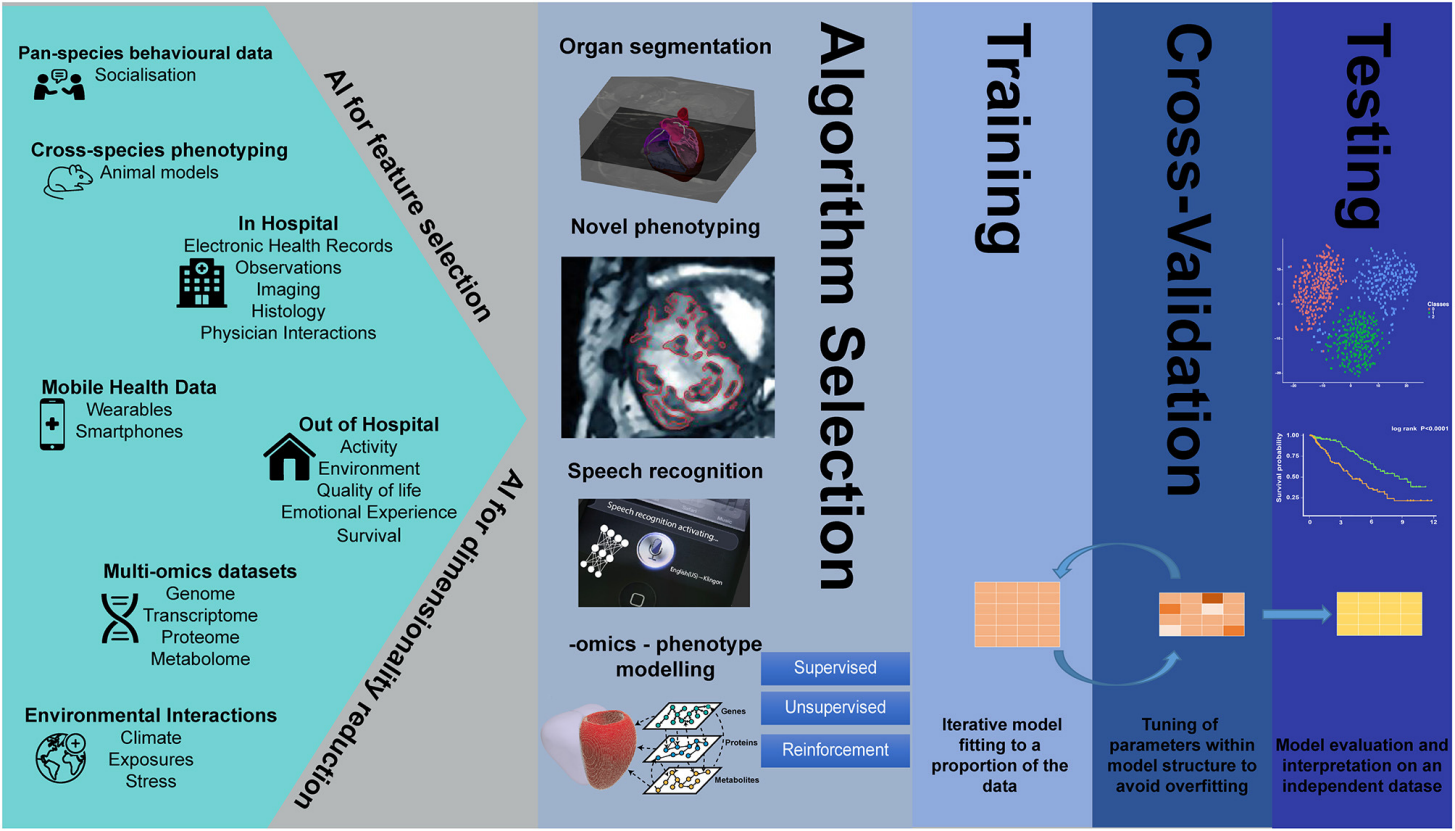
Artificial intelligence in big data imaging-genetics research.

Despite the parallel successes of AI in the fields of genetics and imaging analysis, integrated imaging-genetics research is still an emerging field. However, several studies have already demonstrated the usefulness of AI tools in the analysis of large biological, imaging, and environmental data, in such tasks as dimensionality reduction and feature selection, speech recognition, clustering, image segmentation, natural language processing, variable classification, and outcome prediction (Figure 1).

To predict which dilated cardiomyopathy patients responded to immunoglobulin G substitution (IA/IgG) therapy, as assessed by echocardiography, two supervised ML approaches, a random forest analysis and a support vector machine algorithm, were used independently on gene expression data derived from 48 endomyocardial biopsies (72). The overlapping set of 4 genes that was identified by both ML approaches was superior to clinical parameters in discriminating between responders and non-responders to therapy. The prediction performance was further improved by adding data on the negative inotropic activity (NIA) of antibodies. A support vector machine classifier, also proved to be extremely helpful in identifying specific proteomic signatures that accurately discriminated between patients with heart failure with reduced ejection fraction (HFrEF) and controls in the absence (73) or presence of chronic kidney disease (74). ML pipelines also often use feature selection to more efficiently process high dimensional phenotypes, distinguishing the most informative features from those that are redundant. For example, an information gain method was used to identify speckle-tracking features able to differentiate athlete's heart from HCM. The combination of three different supervised machine learning algorithms (support-vector machine, random forest, and neural network) trained on this sparser data was then shown to be better at distinguishing the two types of remodeling (ML model sensitivity = 87%; specificity = 82%) than conventional echocardiographic parameters (best parameter was e'—sensitivity = 84%; specificity = 74%) (75).

ML approaches have also been successfully used in the identification of new, useful structures in data. One such study, using a hypothesis-free unsupervised clustering approach, revealed four distinct proteomic signatures with differing clinical risk and survival in patients with pulmonary arterial hypertension (76). ML has similarly been able to identify new sub-phenotypes in heart failure with preserved ejection fraction, classifying subjects into three subgroups associated with distinct clinical, biomarker, hemodynamic, and structural groups with markedly different outcomes (77). Okser et al. used a naïve Bayes classifier in a longitudinal imaging-genetics study of 1,027 young adults to identify a predictive relationship between genotypic variation and early signs of atherosclerosis, as assessed by carotid artery intima-media thickness, which could not be explained by conventional cardiovascular risk factors (78).

Classification problems, such as pixel-wise classification of CMR images, are also particularly suited to supervised classical ML (79, 80) and deep learning approaches (81). These high-resolution representations of whole-heart shape and function can encode multiple phenotypes, such as wall thickness or strain, at each of thousands of points in the model (82). Such high-fidelity models were used in a study aiming to clarify the physiological role of titin-truncating variants (TTNtv), known to be a common cause of DCM but surprisingly also present in ~1% of the general population (83). Mass univariate analyses, adjusted for multiple clinical variables and multiple testing, were carried out at over 40,000 points of a statistical parametric map of 1,409 healthy volunteers. This identified an association between TTNtv positive status and eccentric remodeling, indicating a previously unproven physiological effect of these variants in subjects without DCM. A similar phenotyping approach was used by Attard et al. in 312 patients to elucidate the physiological mechanisms that underpinned reported association between certain metabolites and survival in patients with pulmonary hypertension (84). Univariate regression models including clinical, hemodynamic, and metabolic data were fitted at each vertices of a 3D cardiac mesh. These showed coherent associations between 6 metabolites and right ventricular adaptation to pulmonary hypertension as well as showing that wall stress was an independent predictor of all-cause mortality.

ML algorithms have also shown promise in predicting outcomes, such as imaging surrogates of disease or response to treatment, from complex sets of clinical and genetic variables. For example, to predict the presence or absence of coronary plaques on CT coronary angiography, a gradient boosting classifier was trained on a proteomic assay and identified two distinct protein signatures (85). A subset of these was found to outperform generally available clinical characteristics in the prediction of patients with high risk plaques (AUC = 0.79 vs. AUC = 0.65), while a distinct set outperformed clinical variables in predicting absence of coronary disease (AUC = 0.85 vs. AUC = 0.70). In another study, a combination of random forest and neural network methods were used first to identify the most informative subset of clinical and genomic data and then to predict coronary artery calcium (86). Interestingly, the model trained on SNP data only was highly predictive (AUC = 0.85), and better than models trained on clinical data (AUC = 0.61) and on a combination of genomic and clinical data (AUC = 0.83). Further validation experiments in patients with less severe coronary artery calcium showed poor predictive accuracy suggesting that the models' predictive value is limited to a range of (high) coronary calcium or that the models do not generalize well in the broader population. Schmitz et al. investigated the performance of 15 different supervised machine learning algorithms in predicting positive cardiac remodeling in patients that underwent cardiac resynchronization therapy (CRT) from clinical and genomic data (87). Several of the approaches demonstrated clear overfitting (accuracy ~100%), while the algorithm that was identified as the most useful had a fair performance (accuracy = 83%) in addition to high transparency (predictive features easily identified).

Novel deep learning methods are also starting to make an impact in the imaging-genetics field by enabling unprecedented high-throughput image analysis. For example, DL methods have been able to achieve fully automated analysis of CMRs with a performance that is similar to human experts (88) and permitted the rapid segmentation of 17,000 CMRs that were then used in a GWAS (89). This identified multiple genetic loci and several candidate genes associated with LV remodeling, and enabled the computing of a polygenic risk score (PRS) that was predictive of heart failure in a validation sample of nearly 230,000 subjects (odds ratio 1.41, 95% CI 1.26 – 1.58, for the top quintile vs. the bottom quintile of the LV end-systolic volume).

While the use of AI in cardiovascular imaging-genetics has great potential, the limitations and challenges of AI in genetics (90) and imaging (91) are further amplified by combining these very large data. To date, no methodological approaches have been able to include whole-genome and high-resolution whole-heart phenotypes, without requiring extensive dimensionality reduction, filtering and/or feature selection, possibly introducing errors or biases to the input data. Even when this challenge is dealt with, multiple testing correction will continue to be problematic, with the potential for false positive findings likely to only be reliably addressed with replication studies. In AI imaging-genetics, no single method is universally applicable, and the choice of whether and how to use ML or DL approaches will remain task, researcher and population specific, creating difficulties in the pooling of data and meta-analyses. It should not be forgotten that conventional analysis remains valid and has advantages when data are scarce or if the aim is to assess statistical significance, which is currently difficult using deep learning methods. Issues related to the lack of interpretability (“black box”) of some ML algorithms are less of an issue in imaging analysis, where accuracy of analysis can be visually verified, but very relevant to integrated imaging-genetics analysis or risk prediction, where identifying and explaining the features driving the algorithm's output can be virtually impossible. The tendency to over-fit models to training datasets risks reduction in the performance of the model when applied to new populations. These problems are likely to be exacerbated if new test datasets include subjects with differing genetic or physiological backgrounds, data were acquired using different technical conditions (e.g., different scanners or different genotyping batches) or if the quality of data acquired in the research setting significantly differs from real world data sets. Finally, issues regarding privacy, ownership, and consent over vast amounts of genetic and imaging data and legal and ethical considerations for clinicians using integrated imaging-genetics algorithms will become an ever more relevant topic of debate.

Although the application of AI to imaging genetics-research is still new, these promising methods and findings warrant further extensive validation in independent populations. Fully integrated, end-to-end, imaging-genetics DL approaches are theoretically extremely attractive but as yet untested. To confidently implement AI methods in research and clinical practice, challenges regarding standardization of data acquisition and algorithm development and reporting still need to be overcome. Initiatives such as adapting the Transparent Reporting of a Multivariable Prediction Model for Individual Prognosis or Diagnosis (TRIPOD) recommendations (92) to machine learning research [TRIPOD-ML (93)] are very much welcome. Ultimately, the additive value of AI-driven decision making may require robust multi-center studies and randomized controlled trials (94, 95).

## Future Perspectives

The development of body imaging, the elucidation of inheritance and genetics and the application of statistics to medicine were some of the most important medical developments of the past millennium (96). AI now provides an unrivaled ability to integrate these three aspects in imaging-genetics studies of unprecedented scale and complexity. The increasing variety and capabilities of ML tools at the disposal of researchers provide a powerful platform to agnostically revisit classical definitions of disease, to more accurately predict outcomes and to vastly improve our understanding of the genetic and environmental underpinnings of cardiovascular health and pathology. ML approaches will play an increasing role in every field of cardiovascular research, from genomic discovery and deep phenotyping, to mechanistic studies and drug development. Concerted efforts to improve AI study design, reporting, and collaborative validation will greatly contribute to deliver on the great promise of AI and ultimately improve patient care.

## Author Contributions

AM, TD, and DO'R contributed to the content and writing of this manuscript.

### Conflict of Interest

The authors declare that the research was conducted in the absence of any commercial or financial relationships that could be construed as a potential conflict of interest.

## References

[1] Ritchie H Roser M Causes of Death. Available online at: https://ourworldindata.org/causes-of-death (accessed September 25, 2019).

[2] Kathiresan S Srivastava D. Genetics of human cardiovascular disease. Cell. (2012) 148:1242–57. 10.1016/j.cell.2012.03.001 22424232 PMC3319439

[3] Lander ES Linton LM Birren B Nusbaum C Zody MC Baldwin J. Initial sequencing and analysis of the human genome. Nature. (2001) 409:860–921. 10.1038/35057062 11237011

[4] Wetterstrand KA DNA Sequencing Costs: Data from the NHGRI Genome Sequencing Program (GSP). Available online at: www.genome.gov/sequencingcostsdata (accessed September 25, 2019).

[5] Steinhubl SR Muse ED Topol EJ. The emerging field of mobile health. Sci Transl Med. (2015) 7:283rv3. 10.1126/scitranslmed.aaa3487 25877894 PMC4748838

[6] Ngiam KY Khor IW. Big data and machine learning algorithms for health-care delivery. Lancet Oncol. (2019) 20:e262–73. 10.1016/S1470-2045(19)30149-4 31044724

[7] Bogdan R Salmeron BJ Carey CE Agrawal A Calhoun VD Garavan H. Imaging genetics and genomics in psychiatry: a critical review of progress and potential. Biol Psychiatry. (2017) 82:165–75. 10.1016/j.biopsych.2016.12.030 28283186 PMC5505787

[8] Heinz A Goldman D. Genotype effects on neurodegeneration and neuroadaptation in monoaminergic neurotransmitter systems. Neurochem Int. (2000) 37:425–32. 10.1016/S0197-0186(00)00057-7 10871694

[9] Geisterfer-Lowrance AA Kass S Tanigawa G Vosberg HP McKenna W Seidman CE. A molecular basis for familial hypertrophic cardiomyopathy: a beta cardiac myosin heavy chain gene missense mutation. Cell. (1990) 62:999–1006. 10.1016/0092-8674(90)90274-I 1975517

[10] Herman DS Lam L Taylor MR Wang L Teekakirikul P Christodoulou D. Truncations of titin causing dilated cardiomyopathy. N Engl J Med. (2012) 366:619–28. 10.1056/NEJMoa1110186 22335739 PMC3660031

[11] Marian AJ Belmont J. Strategic approaches to unraveling genetic causes of cardiovascular diseases. Circ Res. (2011) 108:1252–69. 10.1161/CIRCRESAHA.110.236067 21566222 PMC3115927

[12] Evangelou E Warren HR Mosen-Ansorena D Mifsud B Pazoki R Gao H Genetic analysis of over 1 million people identifies 535 new loci associated with blood pressure traits. Nat Genet. (2018) 50:1412–25. 10.1038/s41588-018-0205-x 30224653 PMC6284793

[13] Leon-Mimila P Wang J Huertas-Vazquez A. Relevance of multi-omics studies in cardiovascular diseases. Front Cardiovasc Med. (2019) 6:91. 10.3389/fcvm.2019.00091 31380393 PMC6656333

[14] Houle D Govindaraju DR Omholt S. Phenomics: the next challenge. Nat Rev Genet. (2010) 11:855–66. 10.1038/nrg2897 21085204

[15] Plomin R Haworth CM Davis OS. Common disorders are quantitative traits. Nat Rev Genet. (2009) 10:872–8. 10.1038/nrg2670 19859063

[16] Vasan RS Glazer NL Felix JF Lieb W Wild PS Felix SB. Genetic variants associated with cardiac structure and function: a meta-analysis and replication of genome-wide association data. JAMA. (2009) 302:168–78. 10.1001/jama.2009.978-a 19584346 PMC2975567

[17] Jokerst JV Cauwenberghs N Kuznetsova T Haddad F Sweeney T Hou J. Circulating biomarkers to identify responders in cardiac cell therapy. Sci Rep. (2017) 7:4419. 10.1038/s41598-017-04801-7 28667255 PMC5493650

[18] Stringer S Wray NR Kahn RS Derks EM. Underestimated effect sizes in GWAS: fundamental limitations of single SNP analysis for dichotomous phenotypes. PLoS ONE. (2011) 6:e27964. 10.1371/journal.pone.0027964 22140493 PMC3225388

[19] Bilder RM Sabb FW Cannon TD London ED Jentsch JD Parker DS. Phenomics: the systematic study of phenotypes on a genome-wide scale. Neuroscience. (2009) 164:30–42. 10.1016/j.neuroscience.2009.01.027 19344640 PMC2760679

[20] Schork NJ Genetics of complex disease: approaches, problems, and solutions. Am J Respir Crit Care Med. (1997) 156(4 Pt 2):S103–9. 10.1164/ajrccm.156.4.12-tac-5 9351588

[21] Suinesiaputra A Sanghvi MM Aung N Paiva JM Zemrak F Fung K. Fully-automated left ventricular mass and volume MRI analysis in the UK Biobank population cohort: evaluation of initial results. Int J Cardiovas Imag. (2018) 34:281–91. 10.1007/s10554-017-1225-9 28836039 PMC5809564

[22] Knackstedt C Bekkers SCAM Schummers G Schreckenberg M Muraru D Badano LP. Fully automated versus standard tracking of left ventricular ejection fraction and longitudinal strain the FAST-EFs multicenter study. J Am Coll Cardiol. (2015) 66:1456–66. 10.1016/j.jacc.2015.07.052 26403342

[23] Taylor CA Fonte TA Min JK. Computational fluid dynamics applied to cardiac computed tomography for noninvasive quantification of fractional flow reserve scientific basis. J Am Coll Cardiol. (2013) 61:2233–41. 10.1016/j.jacc.2012.11.083 23562923

[24] Topol EJ. High-performance medicine: the convergence of human and artificial intelligence. Nat Med. (2019) 25:44–56. 10.1038/s41591-018-0300-7 30617339

[25] Stein JL Hua X Lee S Ho AJ Leow AD Toga AW. Voxelwise genome-wide association study (vGWAS). Neuroimage. (2010) 53:1160–74. 10.1016/j.neuroimage.2010.02.032 20171287 PMC2900429

[26] Benjamini Y Hochberg Y Controlling the false discovery rate - a practical and powerful approach to multiple testing. J R Stat Soc B. (1995) 57:289–300. 10.1111/j.2517-6161.1995.tb02031.x

[27] Smith SM Nichols TE. Threshold-free cluster enhancement: addressing problems of smoothing, threshold dependence and localisation in cluster inference. Neuroimage. (2009) 44:83–98. 10.1016/j.neuroimage.2008.03.061 18501637

[28] Biffi C de Marvao A Attard MI Dawes TJW Whiffin N Bai W. Three-dimensional cardiovascular imaging-genetics: a mass univariate framework. Bioinformatics. (2018) 34:97–103. 10.1093/bioinformatics/btx552 28968671 PMC5870605

[29] Silver D Schrittwieser J Simonyan K Antonoglou I Huang A Guez A. Mastering the game of go without human knowledge. Nature. (2017) 550:354. 10.1038/nature24270 29052630

[30] Alansary A Oktay O Li Y Folgoc LL Hou B Vaillant G. Evaluating reinforcement learning agents for anatomical landmark detection. Med Image Anal. (2019) 53:156–64. 10.1016/j.media.2019.02.007 30784956 PMC7610752

[31] Alipanahi B Delong A Weirauch MT Frey BJ. Predicting the sequence specificities of DNA- and RNA-binding proteins by deep learning. Nat Biotechnol. (2015) 33:831–8. 10.1038/nbt.3300 26213851

[32] Bernard O Lalande A Zotti C Cervenansky F Yang X Heng PA. Deep learning techniques for automatic MRI cardiac multi-structures segmentation and diagnosis: is the problem solved? IEEE Trans Med Imaging. (2018) 37:2514–25. 10.1109/TMI.2018.2837502 29994302

[33] Nieuwenhuis S Forstmann BU Wagenmakers EJ. Erroneous analyses of interactions in neuroscience: a problem of significance. Nat Neurosci. (2011) 14:1105–7. 10.1038/nn.2886 21878926

[34] Chen JH Asch SM. Machine learning and prediction in medicine - beyond the peak of inflated expectations. N Engl J Med. (2017) 376:2507–9. 10.1056/NEJMp1702071 28657867 PMC5953825

[35] Finlayson SG Bowers JD Ito J Zittrain JL Beam AL Kohane IS. Adversarial attacks on medical machine learning. Science. (2019) 363:1287–9. 10.1126/science.aaw4399 30898923 PMC7657648

[36] Liu X Faes L Kale AU Wagner SK Fu DJ Bruynseels A A comparison of deep learning performance against health-care professionals in detecting diseases from medical imaging: a systematic review and meta-analysis. Lancet Digital Health. (2019) 1:e271–e97. 10.1016/S2589-7500(19)30123-2 33323251

[37] Shimabukuro DW Barton CW Feldman MD Mataraso SJ Das R. Effect of a machine learning-based severe sepsis prediction algorithm on patient survival and hospital length of stay: a randomised clinical trial. BMJ Open Respir Res. (2017) 4:e000234. 10.1136/bmjresp-2017-000234 29435343 PMC5687546

[38] Rumsfeld JS Joynt KE Maddox TM. Big data analytics to improve cardiovascular care: promise and challenges. Nat Rev Cardiol. (2016) 13:350–9. 10.1038/nrcardio.2016.42 27009423

[39] Lee D Kapoor A Safi A Song L Halushka MK Crawford GE. Human cardiac cis-regulatory elements, their cognate transcription factors, and regulatory DNA sequence variants. Genome Res. (2018) 28:1577–88. 10.1101/gr.234633.118 30139769 PMC6169896

[40] Juhola M Joutsijoki H Penttinen K Aalto-Setala K Detection of genetic cardiac diseases by Ca(2+) transient profiles using machine learning methods. Sci Rep. (2018) 8:9355 10.1038/s41598-018-27695-5 29921843 PMC6008430

[41] Tarroni G Oktay O Bai W Schuh A Suzuki H Passerat-Palmbach J. Learning-based quality control for cardiac MR images. IEEE Trans Med Imaging. (2019) 38:1127–38. 10.1109/TMI.2018.2878509 30403623

[42] de Marvao A Dawes TJ Shi W Durighel G Rueckert D Cook SA. Precursors of hypertensive heart phenotype develop in healthy adults: a high-resolution 3D MRI study. JACC Cardiovasc Imaging. (2015) 8:1260–9. 10.1016/j.jcmg.2015.08.007 26476505 PMC4639392

[43] Bhuva AN Treibel TA De Marvao A Biffi C Dawes TJW Doumou G. Sex and regional differences in myocardial plasticity in aortic stenosis are revealed by 3D model machine learning. Eur Heart J Cardiovasc Imaging. (2019) jez166. 10.1093/ehjci/jez166 31280289 PMC7100908

[44] Sengupta PP Huang YM Bansal M Ashrafi A Fisher M Shameer K. Cognitive machine-learning algorithm for cardiac imaging: a pilot study for differentiating constrictive pericarditis from restrictive cardiomyopathy. Circ Cardiovasc Imaging. (2016) 9:e004330. 10.1161/CIRCIMAGING.115.004330 27266599 PMC5321667

[45] Cikes M Sanchez-Martinez S Claggett B Duchateau N Piella G Butakoff C. Machine learning-based phenogrouping in heart failure to identify responders to cardiac resynchronization therapy. Eur J Heart Fail. (2019) 21:74–85. 10.1002/ejhf.1333 30328654

[46] Sanchez-Martinez S Duchateau N Erdei T Kunszt G Aakhus S Degiovanni A. Machine learning analysis of left ventricular function to characterize heart failure with preserved ejection fraction. Circ Cardiovasc Imaging. (2018) 11:e007138. 10.1161/CIRCIMAGING.117.007138 29661795

[47] Mortazavi BJ Downing NS Bucholz EM Dharmarajan K Manhapra A Li SX. Analysis of machine learning techniques for heart failure readmissions. Circ Cardiovasc Qual Outcomes. (2016) 9:629–40. 10.1161/CIRCOUTCOMES.116.003039 28263938 PMC5459389

[48] Dawes TJW de Marvao A Shi W Fletcher T Watson GMJ Wharton J. Machine learning of three-dimensional right ventricular motion enables outcome prediction in pulmonary hypertension: a cardiac MR imaging study. Radiology. (2017) 283:381–90. 10.1148/radiol.2016161315 28092203 PMC5398374

[49] Weng SF Reps J Kai J Garibaldi JM Qureshi N. Can machine-learning improve cardiovascular risk prediction using routine clinical data? PLoS ONE. (2017) 12:e0174944. 10.1371/journal.pone.0174944 28376093 PMC5380334

[50] Bai W Sinclair M Tarroni G Oktay O Rajchl M Vaillant G. Automated cardiovascular magnetic resonance image analysis with fully convolutional networks. J Cardiovasc Magn Reson. (2018) 20:65. 10.1186/s12968-018-0471-x 30217194 PMC6138894

[51] Zhang J Gajjala S Agrawal P Tison GH Hallock LA Beussink-Nelson L. Fully automated echocardiogram interpretation in clinical practice. Circulation. (2018) 138:1623–35. 10.1161/CIRCULATIONAHA.118.034338 30354459 PMC6200386

[52] Hannun AY Rajpurkar P Haghpanahi M Tison GH Bourn C Turakhia MP Cardiologist-level arrhythmia detection and classification in ambulatory electrocardiograms using a deep neural network. Nat Med. (2019) 25:65–9. 10.1038/s41591-018-0268-3 30617320 PMC6784839

[53] Howard JP Fisher L Shun-Shin MJ Keene D Arnold AD Ahmad Y. Cardiac rhythm device identification using neural networks. JACC Clin Electrophysiol. (2019) 5:576–86. 10.1016/j.jacep.2019.02.003 31122379 PMC6537849

[54] Howard JP Cook CM van de Hoef TP Meuwissen M de Waard GA van Lavieren MA. Artificial intelligence for aortic pressure waveform analysis during coronary angiography. Mach Learn Patient Safety. (2019) 12:2093–101. 10.1016/j.jcin.2019.06.036 31563678

[55] Biffi C Oktay O Tarroni G Bai W De Marvao A Doumou G (eds). Learning Interpretable Anatomical Features Through Deep Generative Models: Application to Cardiac Remodeling. Cham: Springer International Publishing (2018).

[56] Tison GH Sanchez JM Ballinger B Singh A Olgin JE Pletcher MJ. Passive detection of atrial fibrillation using a commercially available smartwatch. JAMA Cardiol. (2018) 3:409–16. 10.1001/jamacardio.2018.0136 29562087 PMC5875390

[57] Bello GA Dawes TJW Duan J Biffi C de Marvao A Howard L. Deep learning cardiac motion analysis for human survival prediction. Nat Mach Intell. (2019) 1:95–104. 10.1038/s42256-019-0019-2 30801055 PMC6382062

[58] Medved D Ohlsson M Hoglund P Andersson B Nugues P Nilsson J. Improving prediction of heart transplantation outcome using deep learning techniques. Sci Rep. (2018) 8:3613. 10.1038/s41598-018-21417-7 29483521 PMC5827028

[59] Angermueller C Parnamaa T Parts L Stegle O. Deep learning for computational biology. Mol Syst Biol. (2016) 12:878. 10.15252/msb.20156651 27474269 PMC4965871

[60] Martens K Hallin J Warringer J Liti G Parts L. Predicting quantitative traits from genome and phenome with near perfect accuracy. Nat Commun. (2016) 7:11512. 10.1038/ncomms11512 27160605 PMC4866306

[61] Swan AL Mobasheri A Allaway D Liddell S Bacardit J. Application of machine learning to proteomics data: classification and biomarker identification in postgenomics biology. OMICS. (2013) 17:595–610. 10.1089/omi.2013.0017 24116388 PMC3837439

[62] Zelezniak A Vowinckel J Capuano F Messner CB Demichev V Polowsky N. Machine learning predicts the yeast metabolome from the quantitative proteome of kinase knockouts. Cell Syst. (2018) 7:269–83 e6. 10.1016/j.cels.2018.08.001 30195436 PMC6167078

[63] Zeng H Edwards MD Liu G Gifford DK. Convolutional neural network architectures for predicting DNA-protein binding. Bioinformatics. (2016) 32:i121-i7. 10.1093/bioinformatics/btw255 27307608 PMC4908339

[64] Liu F Li H Ren C Bo X Shu W. PEDLA: predicting enhancers with a deep learning-based algorithmic framework. Sci Rep. (2016) 6:28517. 10.1038/srep28517 27329130 PMC4916453

[65] Leung MK Xiong HY Lee LJ Frey BJ. Deep learning of the tissue-regulated splicing code. Bioinformatics. (2014) 30:i121–9. 10.1093/bioinformatics/btu277 24931975 PMC4058935

[66] Kelley DR Snoek J Rinn JL. Basset: learning the regulatory code of the accessible genome with deep convolutional neural networks. Genome Res. (2016) 26:990–9. 10.1101/gr.200535.115 27197224 PMC4937568

[67] Zhou J Troyanskaya OG. Predicting effects of noncoding variants with deep learning-based sequence model. Nat Methods. (2015) 12:931–4. 10.1038/nmeth.3547 26301843 PMC4768299

[68] Eraslan G Avsec Z Gagneur J Theis FJ. Deep learning: new computational modelling techniques for genomics. Nat Rev Genet. (2019) 20:389–403. 10.1038/s41576-019-0122-6 30971806

[69] Libbrecht MW Noble WS. Machine learning applications in genetics and genomics. Nat Rev Genet. (2015) 16:321–32. 10.1038/nrg3920 25948244 PMC5204302

[70] Min S Lee B Yoon S. Deep learning in bioinformatics. Brief Bioinform. (2017) 18:851–69. 10.1093/bib/bbw068 27473064

[71] Zou J Huss M Abid A Mohammadi P Torkamani A Telenti A. A primer on deep learning in genomics. Nat Genet. (2019) 51:12–8. 10.1038/s41588-018-0295-5 30478442 PMC11180539

[72] Ameling S Herda LR Hammer E Steil L Teumer A Trimpert C. Myocardial gene expression profiles and cardiodepressant autoantibodies predict response of patients with dilated cardiomyopathy to immunoadsorption therapy. Eur Heart J. (2013) 34:666–75. 10.1093/eurheartj/ehs330 23100283 PMC3584995

[73] Rossing K Bosselmann HS Gustafsson F Zhang ZY Gu YM Kuznetsova T. Urinary proteomics pilot study for biomarker discovery and diagnosis in heart failure with reduced ejection fraction. PLoS ONE. (2016) 11:e0157167. 10.1371/journal.pone.0157167 27308822 PMC4911082

[74] Farmakis D Koeck T Mullen W Parissis J Gogas BD Nikolaou M. Urine proteome analysis in heart failure with reduced ejection fraction complicated by chronic kidney disease: feasibility, and clinical and pathogenetic correlates. Eur J Heart Fail. (2016) 18:822–9. 10.1002/ejhf.544 27220540

[75] Narula S Shameer K Salem Omar AM Dudley JT Sengupta PP. Machine-learning algorithms to automate morphological and functional assessments in 2D echocardiography. J Am Coll Cardiol. (2016) 68:2287–95. 10.1016/j.jacc.2016.08.062 27884247

[76] Sweatt AJ Hedlin HK Balasubramanian V Hsi A Blum LK Robinson WH. Discovery of distinct immune phenotypes using machine learning in pulmonary arterial hypertension. Circul Res. (2019) 124:904–19. 10.1161/CIRCRESAHA.118.313911 30661465 PMC6428071

[77] Shah SJ Katz DH Selvaraj S Burke MA Yancy CW Gheorghiade M. Phenomapping for novel classification of heart failure with preserved ejection fraction. Circulation. (2015) 131:269–79. 10.1161/CIRCULATIONAHA.114.010637 25398313 PMC4302027

[78] Okser S Lehtimaki T Elo LL Mononen N Peltonen N Kahonen M. Genetic variants and their interactions in the prediction of increased pre-clinical carotid atherosclerosis: the cardiovascular risk in young Finns study. PLoS Genet. (2010) 6:e1001146. 10.1371/journal.pgen.1001146 20941391 PMC2947986

[79] Shi W Lombaert H Bai W Ledig C Zhuang X Marvao A Multi-atlas spectral PatchMatch: application to cardiac image segmentation. Med Image Comput Comput Assist Interv. (2014) 17(Pt 1):348–55. 10.1007/978-3-319-10404-1_44 25333137

[80] Bai W Shi W de Marvao A Dawes TJ O'Regan DP Cook SA. A bi-ventricular cardiac atlas built from 1000+ high resolution MR images of healthy subjects and an analysis of shape and motion. Med Image Anal. (2015) 26:133–45. 10.1016/j.media.2015.08.009 26387054

[81] Duan J Bello G Schlemper J Bai W Dawes TJW Biffi C. Automatic 3D Bi-ventricular segmentation of cardiac images by a shape-refined multi- task deep learning approach. IEEE Trans Med Imaging. (2019) 38:2151–64. 10.1109/TMI.2019.2894322 30676949 PMC6728160

[82] de Marvao A Dawes TJ Shi W Minas C Keenan NG Diamond T. Population-based studies of myocardial hypertrophy: high resolution cardiovascular magnetic resonance atlases improve statistical power. J Cardiovasc Magnetic Resonance. (2014) 16:16. 10.1186/1532-429X-16-16 24490638 PMC3914701

[83] Schafer S de Marvao A Adami E Fiedler LR Ng B Khin E. Titin-truncating variants affect heart function in disease cohorts and the general population. Nat Genet. (2017) 49:46–53. 10.1038/ng.3719 27869827 PMC5201198

[84] Attard MI Dawes TJW de Marvao A Biffi C Shi W Wharton J. Metabolic pathways associated with right ventricular adaptation to pulmonary hypertension: 3D analysis of cardiac magnetic resonance imaging. Eur Heart J Cardiovasc Imaging. (2019) 20:668–76. 10.1093/ehjci/jey175 30535300 PMC6529902

[85] Bom MJ Levin E Driessen RS Danad I Van Kuijk CC van Rossum AC. Predictive value of targeted proteomics for coronary plaque morphology in patients with suspected coronary artery disease. EBioMedicine. (2019) 39:109–17. 10.1016/j.ebiom.2018.12.033 30587458 PMC6355456

[86] Oguz C Sen SK Davis AR Fu YP O'Donnell CJ Gibbons GH. Genotype-driven identification of a molecular network predictive of advanced coronary calcium in ClinSeq(R) and Framingham Heart Study cohorts. BMC Syst Biol. (2017) 11:99. 10.1186/s12918-017-0474-5 29073909 PMC5659034

[87] Schmitz B De Maria R Gatsios D Chrysanthakopoulou T Landolina M Gasparini M. Identification of genetic markers for treatment success in heart failure patients: insight from cardiac resynchronization therapy. Circ Cardiovasc Genet. (2014) 7:760–70. 10.1161/CIRCGENETICS.113.000384 25210049

[88] Bhuva AN Bai W Lau C Davies RH Ye Y Bulluck H. A multicenter, scan-rescan, human and machine learning CMR study to test generalizability and precision in imaging biomarker analysis. Circ Cardiovasc Imaging. (2019) 12:e009214. 10.1161/CIRCIMAGING.119.009759 31547689

[89] Aung N Vargas JD Yang C Cabrera CP Warren HR Fung K. Genome-wide analysis of left ventricular image-derived phenotypes identifies fourteen loci associated with cardiac morphogenesis and heart failure development. Circulation. (2019) 140:1318–30. 10.1161/CIRCULATIONAHA.119.041161 31554410 PMC6791514

[90] Boyle EA Li YI Pritchard JK. An expanded view of complex traits: from polygenic to omnigenic. Cell. (2017) 169:1177–86. 10.1016/j.cell.2017.05.038 28622505 PMC5536862

[91] Petersen SE Abdulkareem M Leiner T. Artificial intelligence will transform cardiac imaging-opportunities and challenges. Front Cardiovasc Med. (2019) 6:133. 10.3389/fcvm.2019.00133 31552275 PMC6746883

[92] Collins GS Reitsma JB Altman DG Moons KG. Transparent reporting of a multivariable prediction model for individual prognosis or diagnosis (TRIPOD): the TRIPOD statement. The TRIPOD Group. Circulation. (2015) 131:211–9. 10.1161/CIRCULATIONAHA.114.014508 25561516 PMC4297220

[93] Collins GS Moons KGM. Reporting of artificial intelligence prediction models. Lancet. (2019) 393:1577–9. 10.1016/S0140-6736(19)30037-6 31007185

[94] Dawes TJW Bello G O'Regan DP Multicentre Study of Machine Learning to Predict Survival in Pulmonary Hypertension. OSF. Available online at: 10.17605/OSF.IO/BG6T9 (accessed Septmeber 25, 2019).

[95] Lin H Li R Liu Z Chen J Yang Y Chen H. Diagnostic efficacy and therapeutic decision-making capacity of an artificial intelligence platform for childhood cataracts in eye clinics: a multicentre randomized controlled trial. EClinicalMedicine. (2019) 9:52–9. 10.1016/j.eclinm.2019.03.001 31143882 PMC6510889

[96] Looking back on the millennium in medicine. N Engl J Med. (2000) 342:42–9. 10.1056/NEJM200001063420108 10620649

